# Oral Antibacterial Drug Prescribing in Primary Care Out-of-Hours Services: A Scoping Review

**DOI:** 10.3390/antibiotics14010100

**Published:** 2025-01-16

**Authors:** Sarah Khalid Al Hussain, Rhian Deslandes, Deborah Edwards, Karen Louise Hodson

**Affiliations:** 1 School of Pharmacy and Pharmaceutical Sciences, Cardiff University, Cardiff CF10 3NB, UK; deslandesre@cardiff.ac.uk (R.D.); hodsonkl@cardiff.ac.uk (K.L.H.); 2 College of Clinical Pharmacy, King Faisal University, Hofuf 31982, Saudi Arabia; 3 Wales Centre for Evidence Based Care, Cardiff University, Cardiff CF14 4XN, UK; edwardsdj@cardiff.ac.uk

**Keywords:** antibiotics, antimicrobial stewardship, after-hours care, out-of-hours, prescribing, primary care, antimicrobial resistance

## Abstract

Background/Objectives: The rapid spread of antimicrobial resistance (AMR) presents a critical threat to global health. Primary care plays a significant role in this crisis, with oral antibacterial drugs among the most prescribed medications. Antibacterial prescribing rates are often high and complicated in out-of-hours (OOH) services, including weekdays outside regular hours, weekends, and holidays, potentially exacerbating AMR. This review aims to identify the existing literature on oral antibacterial drug prescribing within primary care OOH services. Methods: This review followed established frameworks, adhered to PRISMA-ScR guidelines, and the protocol was registered on Open Science Framework. Seven databases were searched from 2017 to May 2022. Data were summarised, tabulated, and presented narratively to explore themes and patterns that aligned with the review objectives. Results: The search identified 28 studies from nine high-income countries, mainly the UK (n = 6) and Belgium (n = 5). Most were quantitative studies (n = 23). Key areas identified included common oral antibacterial prescriptions, prescribing trends, presentations and conditions managed in OOH services, factors and predictors associated with prescribing, prescribing appropriateness, the impact of interventions on prescribing, prescribing in the context of COVID-19, patient satisfaction and expectations, and the challenges encountered, such as factors influencing prescribing behaviour and decision making, safety netting, and communication. Conclusions: This review highlights key areas around oral antibacterial prescribing in primary care OOH services. Despite the numerous articles identified covering various areas within OOH services, the variability in OOH services approaches across countries and studies complicates the comparison of practice. Further research is needed to better understand practices in these settings.

## 1. Introduction

Overprescribing and misuse of antibacterial drugs are primarily linked to the rapid emergence and spread of antimicrobial resistance (AMR) in both developed and developing nations. The rising threat of AMR, together with the lack of newly discovered generations of antibacterial drugs and the scarcity of available effective options against resistant pathogens, poses a serious danger to human health and the global economy [1]. Although AMR has emerged as a clinical concern within secondary care settings, infections caused by resistant pathogens have become prevalent in primary care and community settings in recent years, even in patients with no history of a previous hospital stay [2]. It has been reported that about four-fifths of antimicrobial prescribing takes place in general practices [3], and over one-fifth of antimicrobial prescribing in primary care is considered inappropriate [4]. Literature shows that antibacterial drugs are the most commonly prescribed among all antimicrobial drugs [5], with oral formulations contributing more to AMR than topical forms [6]. Therefore, reducing unnecessary prescribing in these settings has become a fundamental pillar of global antimicrobial stewardship (AMS) initiatives aimed at effectively combating AMR.

An essential part of primary care is the delivery of out-of-hours (OOH) services to individuals in need. These services refer to the extended provision of healthcare outside regular working hours during weekdays, all weekends, and public holidays [7]. It has been claimed that prescribing antibacterial drugs tends to be higher in OOHs than during standard working hours [8].

Despite the growing number of publications focusing on antibacterial prescribing, resistance, and stewardship programmes, most of these have been carried out within normal working hours. It remains unclear what evidence is currently available on prescribing oral antibacterial drugs in primary care OOH services, with limited insights on prescribing patterns, views, experiences, and behaviour of clinicians and/or patients involved in these settings. Although Hart and Phillips published a literature review with a similar focus on OOH antimicrobial prescribing, their literature search was carried out in 2017 using only five broad search terms [9]. These terms were only described as: “antibiotic prescribing”, “antimicrobial prescribing”, “out-of-hours”, “pre-hospital”, and “after-hours care”. A preliminary search of the literature did not identify any further reviews in this topic area, highlighting a need for an updated and comprehensive review. Therefore, this scoping review aims to systematically uncover and map the existing evidence around oral antibacterial drug prescribing in OOH services, using an up-to-date, more detailed, and comprehensive search strategy with extensive search terminologies to capture the most current literature. Given the increasing number of publications and the rising concerns of AMR, this scoping review will be of value in providing insights into how these unique settings may contribute to AMR trends and AMS activities. Furthermore, it will help identify opportunities and key areas for improvement in prescribing practices within OOH services, contributing to the broader effort to combat AMR.

## 2. Results

### 2.1. Search Results

**Figure 1:**
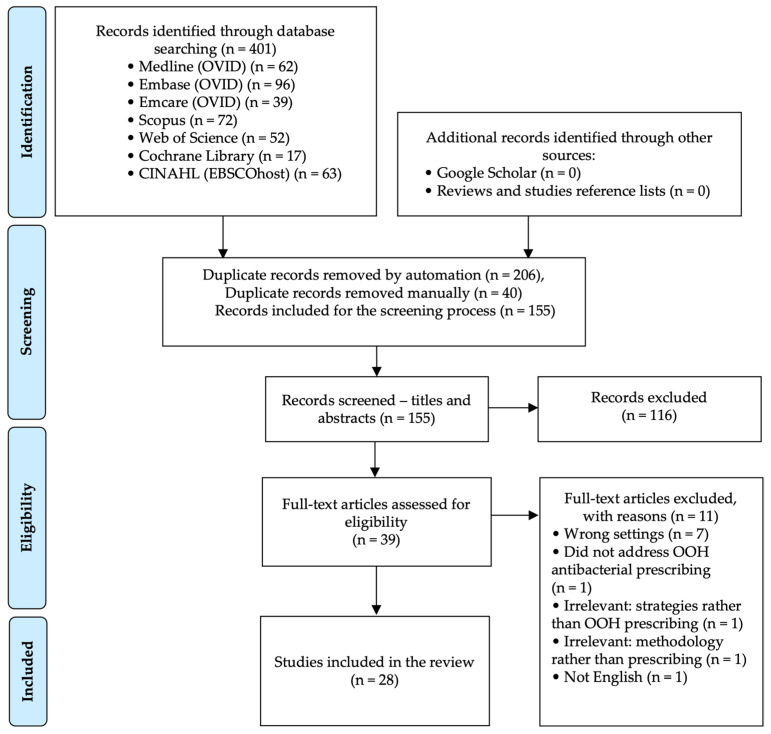
PRISMA flow diagram of search results and selection process of the studies.

Overall, 401 citations were retrieved from the electronic database searches, of which 246 were duplicates. Screening of the titles and abstracts was carried out on 155 records, where 116 citations were excluded. A total of 39 articles were eligible for full-text screening, of which 28 studies were included in the review. The reasons for excluding articles are reported in Figure 1.

### 2.2. Study Characteristics

Included studies originated from nine high-income countries, with the UK accounting for the majority (six studies, 21.4%) [10,11,12,13,14,15], followed by Belgium (five studies, 17.9%) [16,17,18,19,20], Norway [21,22,23,24], Ireland [25,26,27,28], and the Netherlands [29,30,31,32] (four studies each, 14.3%), Australia (two studies, 7.1%) [33,34], and lastly from Iceland [35], Sweden [36], and Denmark [37] (one study each, 3.6%). These studies were disseminated between 2017 and 2022, and the frequency of publication was the highest in 2020 (eight studies, 28.6%) [10,13,19,27,33,35,36].

Literature on oral antibacterial prescribing in primary care OOH services came mostly from quantitative studies (23 studies, 82.1%) [11,12,14,16,17,21,22,23,24,25,26,27,29,30,31,32,33,34,35,36,37], with only a few qualitative studies (five studies, 17.9%) [15,18,19,20,28]. Five of the quantitative studies focused on paediatrics [13,24,25,29,37], one on adults [32], and the remaining lacked age-specific criteria. Qualitative studies explored the prescribers’ views and antibacterial prescribing practices working in OOH, such as their decision making and challenges encountered [15,18,19,20,28]. Although oral antibacterial drug prescribing and practice were reviewed in all included articles, it was not the focus of five studies where other medication classes were examined [10,13,33,34,37]. Several studies focused on specific conditions only, such as respiratory conditions (seven studies, 25%) [15,18,23,24,25,26,28], urinary tract infections (UTIs) (two studies, 7.1%) [22,32], gastroenteritis (one study, 3.6%) [21], and fever (one study, 3.6%) [29]. Detailed summaries of the studies are provided in Supplementary Materials Table S1.

### 2.3. Common Prescriptions in OOH Services

Eleven studies examined oral antibacterial prescriptions in OOH services to varying extents and in different ways. Four of these studies measured prescribing in all OOH contacts and identified that antibacterial drugs were the most prescribed class [10,13,34,37], with two reporting they accounted for over half of all prescriptions [13,34]. Seven studies reported on the most prescribed antibacterial drugs [10,14,23,24,29,35,37], highlighting the group of penicillins as the predominant class despite the variations among countries in their preferred antibacterial drugs for specific conditions [10,14,23,24,29,35,37]. Amoxicillin was the most commonly prescribed in the Netherlands [29], and England [10], while penicillin V was preferred for respiratory tract infections (RTIs) in Norway [23,24]. In Denmark, both penicillin V and amoxicillin were commonly prescribed compared to other oral antibacterial drugs [37]. Amoxicillin-clavulanic acid was prescribed the most in Iceland, followed by amoxicillin and penicillin V [35].

Seven studies explored OOH antibacterial prescriptions in relation to in-hours (IH) services using different approaches to report their findings. Five studies reported that OOH patients were more likely to receive antibacterial prescriptions [22,30,33,34,36], and one revealed a higher percentage of broad-spectrum drugs prescribed compared to IH services [11]. One of these studies from the Netherlands found that amoxicillin, nitrofurantoin, and amoxicillin-clavulanic acid were prescribed more frequently during OOH services [30]. However, findings from a qualitative interview study reported that, despite the lower prescribing threshold in OOH services among prescribers, their choice of antibacterial drug was similar to IH settings [20]. This was supported by Cronberg et al., whose quantitative study showed higher prescribing in OOH services but comparable choices of antibacterial drugs for each diagnosis across both services [36].

### 2.4. Conditions Presented and Managed in OOH Services

Eight studies on OOH services identified common conditions, including respiratory, urinary, ear, and skin-related infections, presenting to the services, with varied prevalence across countries [13,22,26,30,33,34,36,37]. Nonetheless, these studies explored prescribing for the different clinical areas, either examining a wide range of conditions [13,30,33,34,36,37] or focusing on specific ones, such as UTIs [22] and respiratory-related ailments [26]. Three of these studies compared the prevalence of conditions between IH and OOH services [30,33,36].

Eight studies also revealed variability in prescribing patterns of antibacterial drugs across medical conditions and settings to varying degrees [10,13,16,23,24,35,36,37]. Certain diagnoses were associated with higher prescription rates in comparison to others; for instance, one study found sinusitis and bronchitis as the most common conditions for which antibacterial drugs were prescribed [35], while another reported that the highest frequency of antibacterial prescriptions was attributed to UTIs followed by respiratory conditions [10].

### 2.5. Factors and Predictors Correlated with Antibacterial Prescribing

Among the studies reviewed, four examined factors and predictors of antibacterial prescribing [23,24,25,32]; three focused on respiratory-related conditions [23,24,25], while one study specifically investigated UTIs [32]. Overall, these studies identified different prescribing predictors related generally to the patient’s presenting symptoms and diagnoses (e.g., positive ear findings, no vomiting, parents beliefs [24], tonsillitis or sinusitis [23]), laboratory test results (e.g., presence of nitrite, leukocytes, or erythrocytes [32] or elevated C-reactive protein (CRP) level [24]), the duration of consultations (e.g., short consultations [23]), and presenting to OOH care, which was associated with higher prescribing odds [25].

### 2.6. Appropriateness of Prescribing Within OOH Services

Four studies investigated the quality of antibacterial prescribing within primary care OOH through either adherence to guidelines [25,30,32] or antibiotic prescribing quality indicators (APQIs) [16]. Overall, these studies highlighted variations in the appropriateness of prescribing antibacterial drugs, with some conditions (e.g., cystitis, otitis media [16,30], tonsillitis, and impetigo [30]) demonstrating higher adherence than others. Additionally, antibacterial prescribing during OOH telephone consultations was more guideline-compliant than in GP office visits [32], and delayed prescribing adhered more to guidelines than immediate prescribing in both IH and OOH services [25].

### 2.7. Impact of Interventions on Antibacterial Prescribing Within OOH

Two studies were identified where specific interventions were developed and implemented to improve prescribing within primary care OOH services [27,29]. The interventions were mainly educational, including an interactive booklet as a guide to facilitate interactive discussion between clinicians and parents [29] as well as electronic pop-up messaging classifying antibacterials into red (avoid) and green (preferred) with other supporting materials [27]. These studies demonstrated that such interventions could have a positive impact on antibacterial prescribing within OOH services, resulting in more appropriate and guideline-concordant prescribing practices.

### 2.8. Trends of Antibacterial Prescribing

Six studies investigated trends in prescribing within OOH services across different countries [11,12,14,22,25,36]. These studies examined prescribing patterns, seasonal variations, and changes in prescribing practices for various conditions. Antibacterial drug prescribing in OOH settings generally declined over the years in Sweden from 2006 to 2014, especially in children and those with RTIs [36]. For both IH and OOH services in England, antibacterial drug prescribing peaked each December [12,14] and dropped in July [12]. Additional studies conducted in England revealed that the proportion of broad-spectrum antibacterial prescribing was highest each year in July [12] and August [14] for both services, with OOH services contributing to higher broad-spectrum prescribing than IH services [11,12,14].

In Ireland, antibacterial drug prescribing rates among children with upper RTIs were reduced following the introduction of free GP services in July 2015, both in daytime and OOH services [25]. While in Norway, antibacterial drug prescribing for UTIs increased over the study period from 2006 to 2015 within IH and OOH services, with about 52% of cases in primary care resulting in an antibacterial prescription [22]. Within England’s OOH services [14], trimethoprim prescribing and the trimethoprim-to-nitrofurantoin ratio declined between 2016 and 2020 [14], with consistently shorter UTI prescription durations reported in a study of prescribing from 2010 to 2017 compared to IH services [11].

#### Prescribing in the COVID-19 Context

Three studies explored the change in the prescribing of antibacterial drugs following the COVID-19 lockdown in terms of overall trend, volume of specific drugs or conditions, and mode of contact, with a common trend of reduction observed [14,17,31]. In Belgium, a drop of 42.9% in OOH antibacterial prescribing was seen, with a decrease in amoxicillin and amoxicillin-clavulanic acid prescribing, while nitrofurantoin remained stable. Both telephone and face-to-face prescribing decreased by 56.5%, with face-to-face contacts decreasing by about a third after the lockdown and telephone contacts rising [17]. OOH prescribing levels in England were stable before the onset of COVID-19 but started to decrease in March 2020, with increasing proportions of broad-spectrum prescribing in both IH and OOH services. Amoxicillin-clavulanic acid and doxycycline peaked between March and May 2020, unlike previous seasonal trends [14]. In the Netherlands, antibacterial drug prescribing drastically fell during the COVID-19 pandemic with fewer patient encounters during daytime and OOH services but returned to pre-pandemic levels in OOH services in 2021 [31]. Dutch prescribing rates changed considerably for RTIs and children under 11, while UTI prescribing did not, with no long-term impact on prescribing observed [31].

### 2.9. Patients Satisfaction and Expectations When Visiting OOH Care

Two studies mentioned satisfaction with the experience and care provided in OOH [29,37]. Parents generally reported feeling positive, reassured, and satisfied with the care their children received [29,37]. However, one of these studies noted that dissatisfaction was linked to the low rate of antibacterial prescribing in OOH services [37].

In terms of expectations, a study focusing on acute RTIs in OOH care found that patients mainly expected further examination, reassurance, information, and cough medications [26]. While over half of those surveyed were unsure about needing an antibacterial prescription, 34% expected one, especially those with earachea or sore throata, attending subsequent consultations, or eligible for free care. Male patients, however, were less likely to expect a prescription [26].

### 2.10. Challenges Within OOH Services

#### 2.10.1. Factors That Influence Antibacterial Prescribing Behaviour and Decision Making

Factors influencing antibacterial drug prescribing decisions among prescribers within OOH services were explored in four qualitative studies [15,19,20,28]. These studies delved into various aspects related to organisation, work environment, patient factors, and professional identity, highlighting the complexities of prescribing in OOH settings. Prescribers often face pressure when prescribing with limited consultation time, mainly for unknown patients, making it more challenging to have a proper discussion to fully assess their actual needs for an antibacterial drug [15,20,28]. It was also perceived that patients attending OOH care are often sicker, increasing the likelihood of prescribing [15,20]. Additionally, prescribers felt pressured to meet expectations [20,28], particularly for those paying for the service [28], and faced additional barriers such as language and cultural differences and those who had already consulted their GPs [20]. Several other factors were also reported that might lead to management uncertainty, such as inability or limited access to patient records, lack of OOH re-access or follow-up, patient awareness and perceived anxiety, fear of missing critical conditions, and loyalty toward regular GPs [15,20]. Meeting new patients and building rapport might also influence prescribing behaviour [15]. Prescribers felt they had different duties working OOH [19,20], with extra burden, limited diagnostics, and feeling less connected and more pressured by patients or faster-working peers [20]. Furthermore, the high turnover and varying shifts within the workforce make consistent training and education difficult, contributing to the complexities of antibacterial prescribing behaviour in OOH settings and often leading to prescribing practices that may contradict local guidelines [15].

#### 2.10.2. Communication and Managing Expectations

Effective communication, understanding patients’ expectations, and aligning prescribing decisions with appropriate clinical assessments were highlighted in OOH services. Managing expectations and providing explanations and reassurance enable prescribers to make informed decisions, resulting in enhanced patient satisfaction and outcomes. These were discussed in three studies [15,18,19]. In England, a three-stage approach for communicating management decisions was followed: managing patient expectations of antibacterial drugs, discussing and negotiating the care plan by explaining the severity and course of illnesses, and concluding with safety netting advice on alarming symptoms, side effects, and alternative care options [15]. In Belgium, a study showed that working settings affected prescribers’ communication and decision-making processes [19]. Prescribers’ decisions were influenced by unverified assumptions about patients and the manner in which they framed their concerns rather than the actual details and contents of the complaints [19]. GPs used different communication styles to address expectations, with open-ended communication or with close-ended interaction, which affected their understanding of patients’ ideas, concerns, and expectations (ICE) and, consequently, their prescribing decisions [18]. Addressing patient expectations generally led to straightforward consultations [18].

Prescribers also differed in how they communicated management plans, with a non-antibacterial management plan generally well accepted among patients [18]. It was common for GPs to emphasise that antibacterial drugs were unnecessary following clinical examination and diagnosis. A few GPs reassured patients, addressing concerns, explaining diagnoses, educating them, or managing expectations to justify and support their (non)-antibacterial prescribing plan [19]. Some used assertive and impactful words, such as “conquering infections”, to discourage antibacterial drugs; however, they acknowledged that simpler terms could be more appropriate. When antibacterial drugs were not provided, GPs either offered care advice or prescribed symptomatic relief to meet patient expectations [19].

#### 2.10.3. Safety Netting

Despite the challenges in providing safety netting in OOH services, two studies identified approaches and practices used to ensure that safety netting is in place [15,19]. A watchful waiting approach is often adopted, with GPs highlighting any red flags, although determining the safety of this approach can be difficult [19]. Delayed prescribing is another useful safeguarding tool [19], facilitating treatment and supporting shared decision making [15]. However, the lack of feedback on delayed prescribing and the concerns about patients dispensing against advice might hinder prescribers from issuing such prescriptions [15]. Furthermore, prescribers may increase prescribing on weekends as an additional measure, anticipating limitations in medical coverage the next day [15]. They may also refer patients to their regular GPs, making assumptions about the GPs’ accessibility and response [19].

#### 2.10.4. Differences Between Prescribers’ Experience in OOH Care

One study [15] showed variations in prescribing practices between OOH GPs and nurse practitioners (NPs), with the latter spending longer time with patients, feeling more accountable, and adhering more closely to guidelines compared to GPs, who often prescribe under pressure, and with time constraints, they deviate from guidelines. Both groups acknowledged that GPs manage more complex cases, but NPs viewed inconsistent prescribing as problematic, as patients often linked not receiving an antibacterial prescription with nursing care [15].

#### 2.10.5. Prescribers’ Perspectives on Antibacterial Prescribing and Proposed Solutions

Two studies were identified on OOH prescribers’ perspectives regarding their prescribing and responsibilities toward AMR [15,20]. While prescribers are familiar with local management guidelines and AMR threats, they do not perceive themselves as responsible for the problem [20]. Many GPs do not fully understand their own prescribing profiles and attribute the problem to their peers, expressing a need for feedback for comparison [20]. Additionally, they find it difficult to evaluate the prescribing necessity within OOH, with some proposing the development of setting-specific prescribing guidelines [20]. They also believe that auditing, feedback, and supervision are key to supporting better prescribing practices [15].

## 3. Discussion

This scoping review provides a comprehensive overview of available literature, mapping the evidence from current studies on oral antibacterial prescribing within primary care OOH services. Penicillins were found to be the most frequently prescribed oral antibacterial class across multiple countries. This aligns with findings from Hart and Phillips’ literature review [9], where among the studies they reviewed, Huibers et al. noted beta-lactam-sensitive penicillins as the most prescribed drugs [38], and Hayward et al. reported penicillins as the top prescribed class, with amoxicillin accounting for over 28% of UK primary care prescriptions in IH and OOH settings [39]. Similarly, a study based in Ireland also found penicillins to be the most commonly prescribed class of oral antibacterials in OOH services, with amoxicillin being the most prescribed [40]. In Australian primary care, URTIs were more likely to result in immediate antibacterial prescriptions on weekends and holidays than on weekdays, with narrow-spectrum penicillins being the predominant choice [41]. A study of those presenting with insect bites in OOH services, which accounted for less than 1% of encounters, found that two-thirds of patients were prescribed antibacterial drugs, with flucloxacillin making up 82.1% of antibacterial prescriptions [42]. The high penicillin prescribing rate is possibly attributed to the prevalence of RTI contacts within OOH and primary care as a whole, where penicillins are widely used as first-line treatments [43,44]. Studies included in this scoping review and the literature review by Hart and Phillips [7] consistently identified RTIs as among the most common conditions encountered in OOH services, with a high proportion of antibacterial prescriptions issued for their treatment, as seen in Ireland’s OOH consultations [40]. This aligns with other global systematic reviews [45,46] showing RTIs as a leading cause for seeking care in primary settings, though these findings were not specific to OOH contexts. However, although supported by clinical guidelines for certain conditions, the extensive use of penicillins warrants caution due to rising bacterial resistance. Many pathogens have developed resistance to penicillins, reducing the effectiveness of these drugs. Consequently, healthcare practitioners should adhere to AMS principles and consider alternative treatments when necessary, particularly in cases where the likelihood of resistance is high.

The impact of the COVID-19 pandemic on antibacterial prescribing practices in terms of prescription volumes and mode of consultation was highlighted in this scoping review. Studies included in this review consistently reported an overall decline in prescribed antibacterial drugs during the pandemic for both IH and OOH care in England, Belgium, and the Netherlands [14,17,31], with a significant shift from face-to-face to remote consultations [14]. This aligns with a study in Ireland conducted between December 2019 and 2021, which demonstrated a substantial reduction in OOH prescribing during the early stages of the pandemic, with rates rebounding in 2021 [40]. In contrast, Danish OOH prescribing of these drugs remained relatively stable, showing only a 3% reduction compared to pre-pandemic prescribing [47]. Both studies, however, witnessed a decrease in face-to-face consultations, coinciding with an increase in those conducted remotely [40,47]. This decline could likely be linked to multiple factors associated with the pandemic, including the lockdown measures, social distancing, and reduced infection and transmission rates. Additionally, changes in practitioner-prescribing behaviour and patient health-seeking behaviour during this period may have further influenced these trends. However, despite the overall decline in prescription volumes, it remains unclear whether these changing behaviours and trends persist beyond the pandemic, warranting further investigation to understand the long-term implications of these changes.

Several factors were identified in this scoping review that could influence antibacterial prescribing decisions and behaviours of those working in OOH settings. Many of these factors mirror those described previously in a systematic review by prescribers working within IH services, including diagnostic uncertainty, working under stress, and patient expectations [48]. However, studies within this scoping review identified additional unique challenges specific to OOH care, including the absence of established prescriber–patient relationships, patient anxiety and awareness, parental assessment, busy shifts, limited access to shared medical records, and managing sicker individuals. These challenges, along with limited diagnostic tools, lack of follow-up, and the pressure to meet patient expectations, may lead to unnecessary prescribing. Hart and Phillips’ literature review supported these findings, noting that prescribing decisions could be influenced by parents’ assessment of their child’s condition, either due to the perception that parents have better knowledge about their child’s health or as a way to satisfy parental expectations [9]. Similarly, time pressure was also identified in their review as a common driver that led to less justified prescriptions to end consultations, especially during busy shifts [9]. Additionally, one study found variation in antibacterial prescribing among those working in OOH settings across different consultation types (clinic, telephone, or home visits), with factors such as prescriber familiarity with the settings and their activity levels (e.g., number of patients seen) likely contributing to the pressure they experience and influencing the tendency to prescribe [49].

The need to address factors contributing to unnecessary prescribing in OOH settings was emphasised by the findings of this review. Strategies to mitigate these challenges by promoting long-term health behaviour change could be through creating teachable moments to educate patients as studied by Lawson and Flocke [50] or implementing guideline-based educational outreach to reduce unnecessary prescribing as suggested by Hart and Phillips [9]. A few clinicians working in OOH within this scoping review suggested that tailored guidance suitable for OOH settings, audits, personalised feedback, and supervision could improve prescribing. Broader literature supported the effectiveness of interventions, such as training programmes, outreach, meetings, and lectures, in changing prescribing behaviour, though these have mostly been tested in other settings [51,52,53]. Welsh trainees, for instance, seemed to have positive views about their OOH training [54], suggesting that future training and educational opportunities would be appreciated. Moreover, a systematic review demonstrated that tailored educational outreach, such as addressing specific barriers to change, nearly doubled the likelihood of behaviour change [55]. This suggests that engaging OOH clinicians with interventions designed to meet their specific needs could enhance their effectiveness.

Uncertainty in OOH settings, often driven by factors like the lack of diagnostic tools, further complicates prescribing decisions, as highlighted in this scoping review. This is consistent with findings from a previous study linking the underuse or lack of access to tools, such as CRP testing, to higher prescribing in daytime practices [56]. However, evidence from Danish OOH settings revealed variation in the use of POC testing, including CRP, rapid antigen detection tests (RADT), and urine dipsticks, correlating higher usage with increased antibacterial prescribing [57]. Research also found that such tools support clinicians in making well-informed decisions on whether antibacterial drugs are needed [58,59]. For example, while a reduction in antibacterial prescribing could not be conclusively demonstrated, more than half of clinicians in OOH settings reported changing their prescribing decisions after CRP testing, with nearly two-thirds (64%) changing their decision in cases of suspected lower RTIs [58]. Clinicians also noted that CRP testing supported their decision-making and facilitated communication about not prescribing antibacterial drugs by providing objective measures [58]. In the UK, the NHS-funded point of care (POC) Sore Throat Test and Treat service, initially introduced in several community pharmacies in Wales in November 2018 and has since expanded nationally, helps pharmacists manage uncomplicated throat infections using RADT when necessary. This service shifts management from GPs to pharmacies, many of which operate beyond regular GP hours, contributing to OOH services provision while ensuring antibacterial drugs are given only when warranted. Studies demonstrated the service’s value, reducing unnecessary antibacterial prescriptions, promoting AMS practices, and receiving high satisfaction and positive feedback from both patients and pharmacists [60,61,62,63]. These findings suggest that expanding access to POC testing and diagnostic tools could help address prescribing challenges in OOH settings. However, understanding the service needs and contexts is important to design tailored interventions to maximise the benefits and effective use of these tools.

Prescribers’ thinking and communication skills, as identified in the review, are essential for understanding patients and delivering appropriate management decisions. Enhancing these skills through targeted interventions may ease consultations by fostering effective clinician-patient interactions, leading to improved outcomes. Safety netting advice is a key communication tool clinicians use to provide patients with guidance on managing their conditions and recognising warning signs or deteriorating symptoms. However, a study in Belgian OOH settings found that safety netting advice is often missing or unclear and is rarely documented in medical records [64]. Additionally, clinicians were likely to give more safety netting advice when issuing a (delayed) antibacterial prescription compared to non-prescribing decisions [64]. Such communication gaps might be addressed through initiatives like the programme introduced in England [65]. Based on the OPEN (Out of Hours Prescribing: Enhancing Communication) project, a novel AMS OOH programme was developed to improve communication during OOH consultations for common infections [65]. It includes four 20 min sessions focussing on managing expectations, self-care advice, delayed prescribing, and safety netting [65]. Initiatives of this nature could be shaped, tested, and implemented nationally and globally to strengthen communication and support better prescribing practices.

Although studies from this scoping review showed high antibacterial prescribing within primary care OOH, this does not necessarily imply inappropriate prescribing. For instance, one reviewed study in the Netherlands showed higher prescribing rates in OOH compared to IH services [30], yet the quality of prescribing profiles was comparable. It must be noted, however, that direct comparisons between IH and OOH prescribing remain challenging due to fundamental differences in the clinical environments, available resources, and patient populations in each setting. These differences suggest a need for conducting prescribing quality evaluation within OOH contexts.

Opportunities for improvement in adherence to guidelines and prescribing quality indicators were noted in the review, although studies addressing prescribing quality varied in population, methods, and outcomes. De Man et al. further identified substantial variability among OOH services in antibacterial prescribing for lower UTIs and their prescribing trends over time [66]. Their findings showed that 55% of antibacterial prescriptions adhered to the guidelines’ recommended drugs, with an improvement in adherence from 50.5% in 2016 to 59.8% in 2020 [66]. While some of the reviewed studies showed acceptable levels of adherence to guidelines for specific diagnoses, concerns were raised about inappropriate drug selection of antibacterial drugs and overprescribing for certain infections. These issues are particularly relevant given that antibacterial drug prescribing is often influenced by the severity and nature of infections. For instance, while RTIs were among the most common conditions prompting antibacterial prescriptions, most of these infections, such as sinusitis, sore throat, and otitis media, are typically viral, self-limiting, and occasionally require antibacterial treatment [67,68,69,70], a point also highlighted by Hart and Phillips [9]. It is also possible that the need for timely access to care may also lead to overprescribing, as prescribers may feel compelled to issue an antibacterial prescription to prevent treatment delays, even in situations where such prescriptions are not warranted. This may also result in higher referral rates, especially in severe cases or when further evaluation is needed due to clinical uncertainty in OOH settings. Moreover, many of the reviewed studies assessed prescribing appropriateness retrospectively using routinely collected data, which may have complicated evaluating practices with the lack of accessing detailed patient histories and clinical examinations to understand prescribing urgency for such conditions at the point of prescribing.

Studies in this review revealed that whilst antibacterial drug prescribing in primary care has decreased over time, prescribing in OOH care has either increased or remained stable. This trend may be attributed to AMS interventions and guidance primarily targeting primary care, shifting prescribing to OOH services, as well as the lack of such guidance and training resources tailored to OOH services, as highlighted in Alves et al.’s review [71]. This scoping review also showed a dearth of OOH AMS interventions to support the prudent prescribing of antibacterial drugs, with only two studies evaluating the efficacy of two interventions [27,29]. Both interventions, an interactive prescribing booklet and a simplified educational intervention, proved effective, though the booklet’s success was limited to those who actually used it [27,29]. More recently, the implementation of multifaceted interventions in Belgian OOH settings through participatory action research demonstrated promising outcomes [72]. Interventions included e-learning packages for GPs, patient leaflets, printed guideline summaries, electronic pop-ups to guide or withhold antibacterial prescriptions, interdisciplinary meetings with pharmacists, and CRP POC tests alongside usage guidance and prescribing posters [72]. This approach resulted in reduced total prescribing and increased adherence to guideline-recommended choices for RTIs [72]. Another two-year multifaceted intervention in Spanish primary care, including four OOH services, introduced leadership groups, educational sessions, feedback at individual and centre levels, infographics for clinicians and patients with cystitis, and updated local resistance data [73]. While it did not reduce urine culture requests for uncomplicated UTIs, it improved cystitis management, notably increasing the use of appropriate first-line drugs in OOH centres [73]. There is a clear need for AMS interventions and prescribing strategies designed for OOH care, and promoting and evaluating the uptake of these is essential. While employing multiple interventions simultaneously may maximise overall benefits, it may be reasonable to examine each intervention individually to determine its specific impact on prescribing practices.

The studies identified in this review were all from high-income countries. This may reflect the possible challenges faced in low- and low-middle-income countries (LMICs), such as the lack of trained healthcare workers, insufficient infrastructure, and financial limitations, which often limit access to basic emergency services [74]. These challenges suggest that well-established OOH services are likely to be limited or unavailable in many healthcare systems. Enhancing access to both emergency and OOH care could be an important objective for these countries as they work to improve their healthcare delivery.

### 3.1. Implications for Research and Practice

Addressing global concerns about antibacterial drug prescribing and the rising bacterial resistance requires a deeper exploration of prescribing practices, particularly within OOH services. Most research in OOH services has focused on the volume of prescribing, but understanding prescriber and patient behaviours is critical to identify potential factors affecting daily practice across the different countries.

Healthcare professionals in OOH care need to be supported and better resourced to deliver the best care. Developing and evaluating AMS interventions should be a priority within primary care OOH services with high rates of antibacterial prescribing. Although many initiatives exist to optimise the rational use of antibacterial drugs, such as the national prescribing indicators (NPIs) in Wales [75], to the reviewers’ knowledge, none were specific to Wales OOH settings. It is still unclear if interventions designed for primary care apply to OOH care. A paucity of literature on an intervention’s impact on OOH prescribing practices and whether its effect on prescribing or prescriber’s behaviour is sustainable limits clear conclusions on its success.

More research on service improvement and OOH-tailored interventions and strategies to facilitate appropriate prescribing is needed. The findings also encourage developing practical and easy-to-implement interventions that may ensure a high uptake among prescribers and help them adhere, considering the busy environment and time pressure. Standardising study methods and outcomes would allow comparison of intervention effectiveness across studies.

### 3.2. Strengths and Limitations

This review provides a comprehensive exploration of the subject, employing an extensive search across several databases with broad inclusion criteria. Transparency and rigour were ensured by following JBI and PRISMA-ScR guidance where possible in searching, screening, and reporting to ensure reproducibility. It also builds on an earlier review, incorporating current literature into existing knowledge and ensuring completeness of reporting despite the differences across the identified studies. However, the review has a few drawbacks, including the exclusion of non-English studies and those before 2017, which may have led to missing out on relevant information. Additionally, due to the nature of PhD projects, only one reviewer was involved in the data charting and synthesis. However, to mitigate this, two reviewers reviewed 10% of these stages, and any queries were discussed throughout. Synthesising findings and objectively comparing global OOH prescribing practice was challenging due to the heterogeneity of study populations, variations in OOH services implementation across healthcare systems worldwide, and inconsistencies in methodologies and outcomes reporting. Although a few relevant studies were published after the search was completed, these were subsequently identified and incorporated into the discussion, ensuring the review remains as current as possible.

## 4. Materials and Methods

Given that the objective of the review was to map the existing evidence and provide an overview of oral antibacterial drug prescribing in primary care out-of-hours (OOH) services, a scoping review was chosen. This approach is more suitable for broad explorations of a topic, while a systematic review typically addresses narrower, more specific research questions [76]. This review followed Arksey and O’Malley’s methodological framework [77], which was further enhanced by Levac et al. [78], and the guidance of the JBI [79]. The review is reported according to the Preferred Reporting Items for Systematic Reviews and Meta-Analyses extension for Scoping Reviews (PRISMA-ScR) checklist and reporting guideline [80]. The protocol was registered with the Open Science Framework (OSF; registration: https://osf.io/jfscn/, accessed on 13 December 2024) [81].

### 4.1. Research Question/Objective

The objective was to identify available literature on prescribing oral antibacterial drugs in primary care OOH services. The question and inclusion criteria were developed based on the population, concept, and context (PCC) framework [79].

### 4.2. Study Eligibility

Studies were included if they met the following criteria:

- Participants: Healthcare practitioners working in primary care OOH services, including general practitioners (GPs) and non-medical independent prescribers (NMIPs), patients of any age group, or patient carers visiting primary care OOH services.
- Concept: Any aspects related to prescribing oral antibacterial drugs, such as views, behaviour, interventions, trends, and patterns.
- Context: Primary care OOH services worldwide.
- Study design: Published non-grey literature, including primary research of any type (qualitative, quantitative, or mixed methods) and secondary analysis studies.
- Language and time frame: Publications in English from 2017 onwards to capture evidence relevant to the topic following Hart and Phillip’s review search [9], which involved studies from a search conducted prior to 2017.

Studies were excluded if they did not report on oral antibacterial drugs (e.g., topical), were related to other drugs or diseases, were undertaken in secondary or tertiary care, or if primary care OOH data were not reported separately from IH data.

### 4.3. Search Strategy

The search terms from Hart and Phillips’ review [9] were not used due to their limited comprehensiveness, which may have restricted the number of identified studies. Instead, a three-step approach was followed to identify relevant studies [79]. An initial pilot search was undertaken in two electronic databases, Medline and Embase, via OVID, using the terms “after-hours” OR “out-of-hours” AND “antimicrobial prescribing” OR “antibacterial prescribing” to identify relevant search terms. The titles, abstracts, and index terms of the articles identified were screened, and the identified terms were grouped into three concepts, as shown in Table 1. A second comprehensive search was undertaken in May 2022, using those terms in Table 1, with the input of an experienced research librarian in MEDLINE (OVID), Embase (OVID), Emcare (OVID), CINAHL complete (EBSCOhost), Web of Science Core Collection, Scopus, and the Cochrane library. The full search strategy for each database is presented in Supplementary Materials (Tables S2–S7).

Additionally, a broad Google Scholar search was completed to ensure the extensiveness of the search, and the first 50 records of the results were reviewed for inclusion [82], and those meeting the pre-defined criteria were excluded immediately if already identified via the electronic search. The third search step was screening the reference lists of the included studies and excluded reviews to identify studies that were not captured through the electronic database and Google Scholar searches.

### 4.4. Screening and Selection

Search results were imported into EndNote 20^TM^, where duplicates were removed automatically and manually by one reviewer (SA). The remaining citations were exported into a Microsoft Excel^TM^ spreadsheet for the screening process. The titles and abstracts of the resulting records were screened independently by three reviewers against the predefined criteria for inclusion, where one reviewer (SA) screened all records and two other reviewers (KH, RD) each screened half. Any disagreement between reviewers was resolved through discussion to reach a consensus. The second screening step, which involved full-text screening of relevant citations, was completed by one reviewer (SA). When queries arose, these were discussed with the other two reviewers (KH and RD), and decisions were made by consensus.

### 4.5. Data Extraction

A data extraction form was adapted from the JBI [79] and customised to extract relevant study data into a Microsoft Excel^TM^ spreadsheet. The form was first piloted on 10% of included studies by one reviewer (SA) [83] and then reviewed by two others (KH and RD). The form was revised as needed until a consensus was achieved. The data charting was completed by the reviewer (SA), and 10% was reviewed jointly by the other reviewers (KH and RD), with whom any queries arising at this stage, whether from the 10% sample or beyond, were discussed and resolved. Extracted data included the author, year, aim, design and methodology, country, population, settings, and key findings related to oral antibacterial prescribing. Because of the scoping review nature, evaluating the methodological quality of the included studies was not applicable [84].

### 4.6. Data Presentation

Following the guidance from Popay et al. (2006), the reviewer (SA) used various tools and techniques—such as textual descriptions, tabulations, groupings, and clusters—to extract summary data from eligible full-text studies [85]. These were tabulated and presented narratively to help explore common themes and patterns across the studies as related to the objectives of the scoping review.

In the literature, the terms “antibiotics” and “antibacterial drugs” are often used interchangeably, although the term “antibiotics” does not necessarily involve synthetic and semisynthetic substances [86]. To maintain consistency and clarity, the review standardised terminology using the term “antibacterial drugs” throughout, replacing “antibiotics” where necessary, even when the original studies used the latter term. Furthermore, the use of “antibacterial drug(s)” in this review refers specifically to systemic oral formulations, distinguishing them from other forms such as topical or other routes of administration. When summarising the results, the term “antibacterial drug” was consistently used to specifically reference oral formulation.

## 5. Conclusions

This scoping provides an overview of the research on primary care OOH services addressing different aspects of oral antibacterial drug prescribing. Enhancing the antibacterial drug prescribing practice to support the rational consumption of antibacterial drugs is necessary. The findings also reinforce the significance of establishing AMS programmes at national and international levels to reduce inappropriate prescribing within OOH care, which would positively impact both healthcare systems and human health. Stakeholders and policymakers could develop multifaceted interventions and strategies to promote behavioural changes and mitigate inappropriate prescribing within OOH care.

Despite the satisfactory number of studies identified, the different OOH approaches across countries and the heterogeneous methods and outcomes may confound proper comparisons of practice. Future research could build upon this review to better understand current practice in these settings and their contribution to AMR and AMS efforts. 

## Figures and Tables

**Table 1:** Search terms.

**Concept 1: Out-of-Hours Search Terms**	(Searched with AND)	**Concept 2: Antimicrobials Search Terms**	(Searched with AND)	**Concept 3: Prescribing Search Terms**
(Searched with OR)		(Searched with OR)		(Searched with OR)
Pre-hospital * Prehospital * Out of hours After hours After-hour care / Outside of normal working hours		Antimicrobial * Antibiotic * Antibacterial agents /		Prescrip * Prescrib *

*: Captures all word variations; /: Indicates terms from a database’s controlled index.

## Data Availability

No new data were created or analysed in this study. Data sharing is not applicable to this article.

## Supplementary Materials

The following supporting information can be downloaded at: https://www.mdpi.com/article/10.3390/antibiotics14010100/s1, Table S1: Description of included studies; Table S2: Medline search; Table S3: Embase search; Table S4: Web of Science search; Table S5: Emcare search; Table S6: Scopus search; Table S7: Cochrane library search; Table S8: CINAHL search.
