# Supplementary material for: Oral Antibacterial Drug Prescribing in Primary Care Out-of-Hours Services: A Scoping Review
Source: Antibiotics (Basel). 2025 Jan 16;14(1):100. doi: 10.3390/antibiotics14010100 (PMC11762489; doi:10.3390/antibiotics14010100)
Supplement: Supplementary file 1 [file antibiotics-14-00100-s001.zip › antibiotics-3407244-supplementary.pdf]

antibiotics

MDPI

Supplementary Materials

Description of included studies

Table S1. Description of included studies (alphabetically organised).

Author(s)	Aim(s)	Design	Main Findings
Baker et al. (2020) [33]	• Identify differences in the most common patient reasons for encounter and problems managed in usual hours and after-hours consultations.	• Method: Quantitative: Collected data from a national, cross-sectional study (BEACH study). • Country: Australia. • Duration/year: Apr 2014 – Mar 2015. • Settings: Usual GP hours and after-hours. • Population: Patients of all ages and genders. • Size: 38,275 encounters: 36,529 (95.4%) in usual GP hours and 1746 (4.6%) in after-hours – All encounters and not only antibacterial-related.	• During OOH, patients were more likely to receive antibiotic prescriptions compared to the usual hours. • Significantly higher management rate of acute URTIs, fever, laceration, infections, and injuries during OOH.
Brettell et al. (2020) [10]	• Explore volume and type of prescriptions issued at the end of life. • Explore OOH role in prescribing syringe driver medications.	• Method: Quantitative: A population-based study using OOH electronic record data from Adastra, *clinical* patient management software used by clinicians. • Country: England (Oxfordshire). • Duration/year: 1 Dec 2014 – 30 Nov 2015. • Settings: OOH services. • Population: Palliative patients who had contact with OOH service in the 30 days prior to death • Size: 1530 patients in 2661 encounters, of which 1310 contacts were documented as palliative – All encounters and not only antibacterial-related.	• 319 patients received drug prescriptions, generally not only antibiotics, from the OOH service in their 30 days prior to death. • Morphine was the most commonly prescribed drug overall (13.4%), followed by midazolam (10.4%) and amoxicillin (7.1%). • The most prescribed group of medicines were antibiotics (22.2%). • The most prescribed antibiotics were amoxicillin (30.9%), trimethoprim (25.4%) and co-amoxiclav (17.4%). • 39.7% of antibiotic prescriptions were for UTIs, 31.7% for respiratory conditions and 2.4% for skin infections, 25.4% with no clinical code.
Colliers et al. (2018) [20]	• Assess antibiotics prescribing and dispensing challenges in OOH primary care for GPs and pharmacists. • Identify context-specific elements to help the implementation of behaviour change interventions.	• Method: Qualitative; exploratory study using semi-structured interviews to inform PAR project. Thematic analysis was undertaken. • Country: Belgium. • Duration/year: Not mentioned. • Settings: OOH services (GPCs). • Population: 17 GPs, 5 pharmacists, and 1 GPC manager. • Size: 23 interviews.	• GPs feel antibiotics prescribing threshold in OOH is lower compared to IH. • Differences in GPs' professional identity in OOH (tasks, isolated, insecure, and need to please), type of patients (unknown, vulnerable, or demanding), workload (time-pressured) and lack of diagnostic tools or follow-up. • GPs recognise the antibiotic overprescribing issue, but they do not feel personally responsible for it. • Specific guidelines on antibiotic prescribing, personal feedback, and adequate access to electronic records were suggested.
Colliers et al. (2019) [16]	• Describe antibiotic prescribing in Belgian OOH primary care by indication. • Assess its quality by updating values for ESAC's disease-specific APQI and critically appraise these APQI.	• Method: Quantitative: Collected data from (iCAREdata) database, a research database of linked data on OOH primary care. • Country: Belgium. • Duration/year: 1 Jul 2016 – 30 Jun 2018. • Settings: OOH services in 5 GPCs. • Population: Patients of all ages and genders. • Size: 111,600 encounters (all OOH patient visits); 26,436 antibiotic prescriptions.	• 111,600 encounters resulted in 26,436 (23.7%) antibiotic prescriptions. •The APQI diagnoses (i.e., bronchitis, upper respiratory infection, cystitis, tonsillitis, sinusitis, otitis media, and pneumonia) covered 14,927 (56.7%) prescriptions. • Erysipelas (5.1%) and teeth/gum disease (3.7%) covered more prescriptions than sinusitis or pneumonia. • >75% of tonsillitis patients and >50% with bronchitis, sinusitis, and otitis media were prescribed an antibiotic. • No condition reached the goal (80%-100%) of using the recommended antibiotics. • The choice was near the acceptable range only for otitis media.

			• >10% of patients with bronchitis or pneumonia and >25% of females with acute cystitis received quinolones.<br>• The APQI cover the diagnoses for only 57% of all antibiotic prescriptions.
Colliers et al. (2020) [19]	• Unravel GPs' decision-making during infections consultations in OOH primary care through video-elicitation interviews, with a focus on (non) antibiotic prescribing, using a video consultation as a prompt to explore tacit knowledge, beliefs, attitudes, social influences, and communication that drove their behaviour.	• Method: Qualitative: An elicitation interviews after video-recording consultations. Part of the BAbAR project, which uses PAR approach. Inductive thematic analysis method was used.<br>• Country: Belgium.<br>• Duration/year: Aug 2018 – Nov 2018.<br>• Settings: OOH services.<br>• Population: GPs in OOH services.<br>• Size: 160 consultations were videos-recorded from 21 GPs.	• GPs found that their (non) antibiotic prescribing decision-making was subconsciously influenced by their own interpretation of information.<br>• GPs frequently made assumptions (e.g., encounter reason or antibiotic expectations) without confirming them with patients.<br>• GPs follow a dichotomous thinking process: urgent versus not urgent, viral versus bacterial, antibiotics versus no antibiotics.<br>• Safety netting (wait and see, delayed prescribing) is important but a difficult tool in OOH care, with no patient relationship to follow-up.<br>• GPs used power words (e.g., resistance) to strengthen their message.
Colliers et al. (2021) [18]	• Describe how patients present their problem and how GPs interact with this and elicit the ICE of patients, and in what possible ways this doctor-patient communication relates to antibiotic prescribing for RTIs in OOH care.	• Method: Qualitative; using recorded consultation videos. Part of the BAbAR project, which uses PAR approach to improve antibiotic prescribing quality. Deductive and inductive descriptive analysis.<br>• Country: Belgium.<br>• Duration/year: During the day at weekends; from the end of Aug to Nov 2018.<br>• Settings: OOH services.<br>• Population: Patients with RTIs and GPs managing them.<br>• Size: 77 videos on RTIs from 19 GPs.	• GPs who used patient-centred communication skills learned more about the patient's perspective on the illness.<br>• Various reasons for encounters were reported, such as a sick note need, leaving for upcoming holidays, sent by family members, or reasons related to the illness.<br>• Reason for encounter for some patients was the belief that seeing a GP could change the course of their illness.<br>• While patients ideas, concerns, and expectations frequently remained unstated, they were expressed by choice of words, tone of voice, repetition of words.<br>• In some cases, delayed prescribing was used to address patient unspoken expectations for antibiotics.<br>• A non-antibiotic management plan was well-accepted by patients.
Colliers et al. (2021) [17]	• Describe trends in the antibiotic prescribing of GPs for RTIs, before and after the start of the COVID-19 lockdown in Belgian OOH care.	• Method: Quantitative: An observational study using routinely collected health data.<br>• Country: Belgium.<br>• Duration/year: 2019 – 2020.<br>• Settings: OOH services.<br>• Population: Patients of all ages and genders.<br>• Size: 388,293 contacts and 268,430 prescriptions.	• The number of antibiotic prescriptions per weekend per 100,000 population was 42.9% lower among all contacts compared to before the implementation of the COVID-19 lockdown<br>• For antibiotic prescribing per contact, a decrease of 56.5% among all contacts and of 23.2% for face-to-face contacts only<br>• Amoxicillin and Amoxicillin/Clavulanate are the most often used for RTIs.<br>• The reduction in the prescriptions number was more pronounced for cases with respiratory symptoms resembling those of COVID-19, as well as for antibiotics frequently prescribed for RTIs, such as amoxicillin (a decrease of 64.9%) and Amoxicillin/Clavulanate (a decrease of 38.1%)<br>• Nitrofurantoin prescribing trend remained stable before and after the lockdown
Cronberg et al. (2020) [36]	• Describe the trends in antibiotic prescribing over time.<br>• Compare diagnosis-linked prescribing in general and in detail between IH and OOH in the same population.	• Method: Quantitative: A retrospective, descriptive study based on data from KIDPC* database, which contains information on all visits with an infection diagnosis and all antibiotic prescriptions with or without a visit in primary care in Kronoberg County.<br>• Country: Sweden.<br>• Duration/year: 2006 – 2014<br>• Settings: IH and OOH services<br>• Population: Patients of all ages and genders.<br>• Size: On average, 434 visits (IH: 382 and OOH: 51) and 242 prescriptions (IH: 209 and OOH: 33) per 1000 inhabitants per year.	• During OOH, a reduction both in infection visits (from 65 in 2006 to 43 in 2014, mean annual change: – 3) and antibiotic prescribing (from 43 in 2006 and 26 in 2014, mean annual change: – 2.2) per 1000 inhabitants. The decrease was most notable among children and for RTIs.<br>• When comparing antibiotic prescribing in OOH to IH, the prescription rate was higher in OOH, with the difference being small but significant after adjusting for age, sex, and diagnosis. The reduction after adjustment was explained by a higher visit rate for infections requiring antibiotics (acute otitis media, pharyngotonsillitis, and lower UTIs) in OOH settings.<br>• The choice for antibiotics for common diagnoses was comparable in IH and OOH.
Curtis et al. (2018) [11]	• To describe antibiotic prescribing	• Method: Quantitative: Using 2 sources of publicly available, routinely collected	• Since 2010, the overall rate of antibiotic prescribing has decreased by 18% in primary care, with the sharpest decline after 2013.

	trends in NHS England primary care for the years 1998–2017 using various measures, investigating trends and variation between practices and geographical areas, OOH prescribing, and seasonality.	NHS data: monthly practice-level prescribing data and annual Prescription Cost Analysis data.<br>• Country: England.<br>• Duration/year: For OOH: Oct 2010 – Dec 2017.<br>• Settings: 8052 Standard practice and 260 OOH services.<br>• Population: Patients of all ages and genders.<br>• Size: Not reported.	• Total antibiotic items prescribed in OOH services accounted for 3.3% of the total prescribed in both standard and OOH practices.<br>• OOH practices prescribed a higher proportion of broad-spectrum antibiotics and shorter courses for UTIs compared to standard practices.<br>• Cephalosporin prescribing has declined dramatically (OOH -63.6% and standard hours -49.2%).
De Bont et al. (2018) [29]	• Develop and assess the effectiveness of an illness-focused interactive fever booklet for parents on managing children with fever at family physician OOH care.	• Method: Quantitative: A cluster-randomised controlled trial with 2 arms: an illness-focused interactive booklet arm or a care-as-usual arm.<br>• Country: The Netherlands.<br>• Duration/year: Nov 2015 – Jun 2016.<br>• Settings: 20 OOH services.<br>• Population: Children aged 3 months to 12 years and family physician recorded the consultation as a fever related.<br>• Size: 25,355 children<br>Intervention group: 11,945<br>Control group: 13,410.	• Amoxicillin was the commonly prescribed antibiotic (76.1% of all antibiotics).<br>• The booklet was used in 28.5% of 11,945 consultations in the intervention group.<br>• Antibiotic prescribing during the index consultation was not significantly affected by access to the booklet compared to usual care.<br>• Use of the booklet significantly reduced antibiotic prescribing.<br>• Children treated by family physicians with access to the booklet were less likely to receive any drug prescription.<br>• Parents in the booklet group showed a reduced intention to consult again for similar illnesses.
Debet et al. (2017) [30]	• Provide insight into quantity and quality differences in office hours and OOH antibiotic prescribing.	• Method: Quantitative: Using national, routinely collected Dutch data.<br>• Country: The Netherlands.<br>• Duration/year: 2012.<br>• Settings: Office hours and OOH services.<br>• Population: Patients of all ages and genders.<br>• Size: 6,434,640 antibiotic courses (of which 8.4% in OOH).	• Cystitis and acute otitis media presented most often during OOH.<br>• First-choice prescribing was comparable for IH and OOH settings.<br>• Prescribing rates were higher in OOH, with comparatively more amoxicillin and amoxicillin/clavulanic acid than IH.<br>• Overprescribing was comparable between the settings or even lower than for daily practice.<br>• OOH adherence to guideline >80% for acute otitis media, tonsillitis, cystitis, and impetigo.
Edelstein et al. (2017) [12]	• Describe trends and patterns of antibiotic prescribing among OOH providers in England to inform future stewardship interventions.	• Method: Quantitative: Using national primary care prescribing data from NHS BSA.<br>• Country: England.<br>• Duration/year: 2010 – 2014.<br>• Settings: GP IH and OOH services.<br>• Population: Patients of all ages and genders.<br>• Size: Between 1,055,000 and 1,205,000 antibiotic items each year in OOH.	• OOH prescribing represented 5–5.4% of GP prescribing volume between 2010 and 2013, decreasing to 4.5% in 2014.<br>• Broad-spectrum antibiotic prescriptions proportion increased in OOH when it increased in the CCG they operated in.<br>• The proportion of broad-spectrum antibacterial prescriptions in OOH was higher compared with GP, but it decreased both in GP and OOH.<br>• OOH prescribing volume was stable over time and followed seasonal patterns similar to IH GP prescribing: peaked each year in December and was at its lowest in July.
Edwards et al. (2020) [13]	• Characterise the nature, timing, and outcomes of paediatric contacts with OOH GP.	• Method: Quantitative: Using a database created from OOH electronic record system Adastra, *clinical* patient management software used by clinicians.<br>• Country: England (Oxfordshire).<br>• Duration/year: Dec 2014 – Nov 2015.<br>• Settings: OOH services<br>• Population: Paediatric patients<br>• Size: 27,455 encounters (26.69%) made by 18,987 individuals (29.95%) – All encounters and not only antibacterial-related.	• 14,267 (52.0%) individuals contacted OOH GP regarding an infection.<br>• 5656 (58.7%) prescriptions were for antibiotics.<br>• Children aged 1–4 had the highest percentage of infection (59.6%).<br>• Antibacterial drugs were prescribed in 5420 (19.7%) of contacts, most commonly linked with ear, respiratory, and skin conditions.
Emberland et al. (2022) [21]	• Investigate time trends and patient characteristics associated with antibiotic treatment for GE in Norwegian primary care from 2006 to 2015.	• Method: Quantitative: Using data collected from 2 nationwide registries: KUHR, the national registry of reimbursement claims data from both DGP and OOH services, and NorPD, a complete registry of all prescription drugs dispensed from pharmacies in Norway.<br>• Country: Norway.	• Antibiotic treatment was linked to 23,663 (1.8%) of all GE consultations: DGP 19,617 (1.8%) and OOH 4046 (2%).<br>• No difference between genders in the proportions of consultations with antibiotics.<br>• The lowest proportion of consultations with antibiotics was int he age group 0-4 years (1%), increasing with age up to the categories 55-64 and 65-74 years (3%), with more pronounced increase observed in the OOH services.

		• Duration/year: 2006 – 2015.<br>• Settings: DGP and OOH services.<br>• Population: Patients with GE of all age/gender.<br>• Size: 1,279,867 consultations. DGP: 1,081,162 (84.5%). OOH: 198,705 (15.5%).	• CRP test was used in 58.1% of consultations where antibiotics were prescribed and was used more frequently in OOH services compared to DGP, regardless of whether antibiotics were prescribed.
Haugom et al. (2021) [22]	• Investigate time trends in antibiotic treatment following consultations for UTI in primary care in Norway.	• Method: Quantitative, observational study based on primary care nationwide data from 2 registries: KUHR, the national registry of reimbursement claims data from both DGP and OOH services, and NorPD, a complete registry of all prescription drugs dispensed from pharmacies in Norway.<br>• Country: Norway.<br>• Duration/year: 2006 – 2015.<br>• Settings: DGP and OOH services.<br>• Population: Patients with UTI of all age/gender.<br>• Size: 2,426,643 UTI consultations: DGP: 1,927,615 (79.4%), OOH: 499,028 (20.6%).	• Most UTI consultations were in DGPs (79.4%) compared to OOH (20.6%).<br>• The annual number of UTI consultations increased by 32.7% where cystitis increased by 33.9% (38.9% DGP and 13.8% OOH) and pyelonephritis increased by 14% (28.4% DGP and 16.4% OOH).<br>• Antibiotics were prescribed in 52.1% of UTIs cases in primary care, and the proportion increased during study period.<br>• DGP cystitis with prescribed antibiotic increased from 36.6% in 2006 to 65.7% in 2015 and from 47% to 69.7% in OOH.<br>• DGP pyelonephritis with prescribed antibiotic increased from 28% in 2006 to 46.6% in 2015 and from 50.6% to 64% in OOH.<br>• OOH consultations were significantly more likely to result in antibiotics compared to DGP for both cystitis and pyelonephritis throughout the study period.
Hek et al. (2022) [31]	• Describe changes in antibiotic prescribing in the different phases COVID-19 in 2020 and 2021 compared to 2019 in DGPs and at OOH.<br>• Describe antibiotic prescribing for the two diagnoses for which antibiotics are most frequently prescribed, RTIs and UTIs.	• Method: Quantitative: A retrospective database study using routine care data.<br>• Country: The Netherlands.<br>• Duration/year: 2019 – 2021.<br>• Settings: DGP and OOH services.<br>• Population: Patients of all ages and genders.<br>• Size: Not reported.	• Significantly fewer antibiotics were prescribed during the COVID-19 pandemic both at DGPs and OOH services after government measures became effective, with reduced number of contacts in both settings.<br>• Prescription rates increased both at DGP and OOH services after restrictions were lifted in 2021, returning to pre-pandemic levels at OOH services, but not in DGP.<br>• Changes in antibiotic prescribing rates were prominent for RTIs and among children up to 11 years old, but not for UTIs.
Lindberg et al. (2017) [23]	• Explore factors that predict antibiotic prescribing and broad-spectrum prescribing for ARTIs in primary care OOH.<br>• Explore if the activity level (both per session and per doctor) was correlated with antibiotic prescribing.	• Method: Quantitative: A retrospective data analysis from 2 units' electronic record systems.<br>• Country: Norway.<br>• Duration/year: 2014.<br>• Settings: 2 OOH services.<br>• Population: Patients with ARTIs of all ages and genders.<br>• Size: 6757 ARTI consultations (16.8% of all consultations), 2310 (34.2%) resulted in antibiotic prescription.	• 34.2% of ARTI consultations resulted in an antibiotic being prescribed.<br>• Penicillin V was prescribed the most (69.9% of cases).<br>• Tonsillitis and sinusitis had the highest antibiotic prescription rate among ARTIs.<br>• Shorter consultation durations were linked higher antibiotic prescription rate, though not for broad-spectrum antibiotics.<br>• Broad-spectrum antibiotic prescribing rate was high in the elderly and for bronchitis and pneumonia.
Lous et al. (2019) [37]	• Describe face-to-face consultations for children aged 0 to 5 years in OOH primary care, specifically the RFEs, the diagnoses recorded by the triaging and treating GPs, and the provided care in terms of dispensed prescriptions, reason for referral, and parental satisfaction with the contact.	• Method: Quantitative: A population-based cross-sectional study based on collected data and postal questionnaire sent to parents.<br>• Country: Denmark.<br>• Duration/year: Jun 2010 – May 2011.<br>• Settings: OOH services.<br>• Population: Preschool children (0-5 years) with face-to-face contact.<br>• Size: 2363 contacts:<br>Clinic: 1875; Home visit: 488.<br>Parents: 1220 (51.5%) returned the questionnaire - All encounters and not only antibacterial-related.	• Non-specific complaints were the most common RFE (40%), followed by respiratory symptoms (23%).<br>• The most common diagnosis group was respiratory tract diseases (41%).<br>• 27% of contacts received a prescription; about three-quarters were antibiotics.<br>• 12% of contacts concerned acute otitis media with antibiotics prescribed in 70% of these.<br>• 38% of contacts were concerning fever, with one-fourth receiving antibiotics.<br>• Only 7.4% of contacts were referred for further evaluation.<br>• Parental satisfaction was generally high, but 7% were dissatisfied, which was linked to lower prescription rates.
Maguire et al. (2018) [25]	• Quantify antibiotic prescription rates and compliance with guidelines for URTIs.	• Method: Quantitative: A cross-sectional study of anonymised consultation data.<br>• Country: Ireland.<br>• Duration/year: 2 weeks from Jan of 2015, 2016, and 2017.	• Patients were 50% less likely to receive an antibiotic prescription after introducing free GP care.<br>• Antibiotic prescribing dropped from 70% to 50% in DGP and from 72% to 60% in OOH services.

	• Analyse factors associated with antibiotic prescription in an under-6 sample attending DGP and OOH GP where the time period examined offers an opportunity to examine changes which may be linked to eligibility for free-of-charge services.	• Settings: 4 DGPs and 2 OOH services.<br>• Population: Children under 6 years presenting with URTIs.<br>• Size: 1007 children:<br>DGP: 385 (38.2%).<br>OOH: 622 (61.8%).	• Patients at OOH services were more likely to receive an antibiotic and less likely to receive a deferred antibiotic.<br>• Compliance with guidelines was higher for deferred prescriptions in both settings.
O'Connor et al. (2019) [26]	• Examine patients presenting with acute URTI in an OOH setting expectation of clinical examination, symptom management, information on their condition, reassurance, antibiotic treatment and other possible options including referral.	• Method: Quantitative: A cross-sectional, single-centre study using paper-based questionnaires.<br>• Country: Ireland.<br>• Duration/year: Oct 2017 – Feb 2018.<br>• Settings: 1 urban OOH service.<br>• Population: Patients with acute URTIs of all ages and genders.<br>• Size: 435 questionnaires filled by patients or their parents/guardians if age <18 years.	• The most common presenting symptoms were cough (72%), throat ache (46%) and common cold (26%).<br>• The most common expectations included further examination (53%), reassurance (51%), information (49%) and medication for cough (47%), with 34% expecting an antibiotic.<br>• Patients with earache (44%) or throat ache (39%) were most likely to expect antibiotics.<br>• While not significant, those eligible for free care more likely to expect antibiotics.
O'Connor et al. (2020) [27]	• Analyse the change in the quality of antibiotic prescribing after the introduction of an educational intervention for GPs and patients using the OOH setting categorising antibiotics into a red (avoid) and green (preferred) panel.	• Method: Quantitative: An observational quality improvement study, baseline data based on retrospective OOH electronic notes analysis.<br>• Country: Ireland.<br>• Duration/year: Week 47 – 7 in 2016/2017 and 2017/2018.<br>• Settings: 2 OOH services.<br>• Population: Patients of all ages and genders.<br>• Size: Not reported; 240 GPs participate in providing OOH service.	• The intervention made categorised antibiotics as red (avoid) and green (preferred).<br>• The intervention improved prescribing quality and was well received.<br>• Red antibiotic prescribing decreased from 44% to 17% following the intervention.<br>• Prescribing of amoxicillin-clavulanic acid, the most prescribed non-first line, fell from 33% to 10% after the intervention.<br>• Green antibiotic prescribing increased by 27% after the intervention.
O'Doherty et al. (2019) [28]	• Investigate why GPs in Ireland continue to prescribe antibiotics for ARTI, despite widely publicised guidelines and evidence of their ineffectiveness.	• Method: Qualitative: An explorative study using semi-structured face-to-face interviews that were digitally audio-recorded and transcribed. Thematic analysis was used.<br>• Country: Ireland.<br>• Duration/year: Jun – Aug 2017.<br>• Settings: General practices.<br>• Population: GPs.<br>• Size: 13 GPs – all have experience in GP and worked in OOH setting – Not specific to OOH.	• GPs reported feeling pressured to prescribe for private patients who pay for the service to meet patient expectations.<br>• Limited duration of consultations, especially with unfamiliar patients, is a major factor influencing prescribing practice.
Palsdottir et al. (2020) [35]	• Describe antibiotic prescriptions in OOH primary care in Reykjavik capital area over a one-year period and analyse them by patient age, gender, and diagnosis.	• Method: Quantitative: A population-based retrospective study using electronic data from the OOH medical registration system.<br>• Country: Iceland.<br>• Duration/year: 1 Jan 2014 – 31 Dec 2014.<br>• Settings: OOH services.<br>• Population: Patients of all ages and genders who received oral antibiotics at OOH.<br>• Size: 25,059 contacts resulted in an oral antibiotic prescription (33% of all contacts).	• High rates of antibiotic prescriptions (oral antibiotic was prescribed in 1 out of 3 consultations) and broad-spectrum antibiotics.<br>• The studied diagnoses (sinusitis, cystitis, pneumonia, acute otitis media, and bronchitis) accounted for 12,850 prescriptions of 17 different oral antibiotics (51.3% of all antibiotic prescriptions.<br>• An antibiotic was most often prescribed for sinusitis and bronchitis (27.4% and 24.9% of total antibiotic prescriptions, respectively).<br>• The most common antibiotic prescribed in total, and for the diagnosis studied, was amoxicillin-clavulanic acid, and followed by amoxicillin.<br>• Amoxicillin-clavulanic acid was most often prescribed for acute otitis media (50% of cases) and those diagnosed with pneumonia (40% of cases).<br>• Amoxicillin was most often prescribed for sinusitis (47% of cases).
Rebnord et al. (2017) [24]	• Identify predictors for antibiotic prescription and referral	• Method: Quantitative: A secondary analysis of a randomised controlled study.	• The antibiotic prescription rate was 23%.<br>• Phenoxymethylpenicillin was commonly used (67% of the cases), followed by amoxicillin (20.4% of cases).

	to hospital in a primary care setting.	• Country: Norway.<br>• Duration/year: Winter seasons from Jan 2013 – May 2015.<br>• Settings: 4 OOH services and 1 paediatric emergency clinic.<br>• Population: Children aged 0–6 years with respiratory symptoms and/or fever.<br>• Size: 397 children.	• CRP value >20 mg/L, findings on ear examination, and parents' assessment that the child has a bacterial infection were significantly associated with antibiotics prescribing.<br>• Vomiting in the past 24 hours was negatively associated with prescription.
Spek et al. (2020) [32]	• Examine the workload, diagnostic work-up and treatment of UTIs in healthy adults during OOH primary care.	• Method: Quantitative: A retrospective observational cohort study using data from electronic patient reports.<br>• Country: The Netherlands.<br>• Duration/year: 2018 – full year.<br>• Settings: 2 OOH services.<br>• Population: Adult patients with UTI symptoms.<br>• Size: 5657 patients.	• An average of 8 patients per day contacted OOH because of UTI symptoms.<br>• 74% of patients who contacted or visited OOH received antibiotics.<br>• 79% of patients with negative nitrite tests received antibiotics.<br>• Men and pregnant patients received fewer antibiotic prescriptions.<br>• Antibiotic prescription rates were unaffected by diabetes mellites or antibiotic prophylaxis use for recurrent UTIs<br>• Positive nitrite, leukocytes, or erythrocytes was a predictor for prescription.<br>• Prescription was not in line with guidelines in > 1 out of 5 patients.
Turner et al. (2017) [34]	• Characterise a typical inner metropolitan after-hours patient cohort in Australia.	• Method: Quantitative: Analysis of data from the MAGNET database, which contains both demographic and clinical information extracted from the computerised medical records for patients that have attended any of the 50 participating GP clinics in the inner east Melbourne metropolitan region.<br>• Country: Australia.<br>• Duration/year: 1 Jan 2005 – 31 Dec 2014.<br>• Settings: After-hour GP.<br>• Population: Patients of all ages and genders.<br>• Size: 46,074 patients; 64,800 encounters – All encounters and not only antibacterial-related.	• Most common diagnoses: respiratory system diseases (13.4%, of which 71.2% RTIs), gastrointestinal diseases (12.6%, of which 48.5% throat infections) and eye and ear problems (11.6%, mostly acute otitis media 77.9% and conjunctivitis 9.9%).<br>• Antibacterial medications accounted for more than half (53.0%) of all prescriptions and contributed to a much higher proportion of prescribing compared to non-after hours (11.9%), with 34% of antibiotics prescribed to patients under 18 years old.
Williams et al. (2018) [15]	• Explore antibiotic prescribing practices of GPs and NPs: Objective I: Identify GP and NP experiences of prescribing antibiotics for RTIs in OOH, to explore facilitators and barriers to reducing antibiotic prescribing. Objective II: Identify similarities and differences between GP and NP antibiotic prescribing.	• Method: Qualitative: Semi-structured telephone interviews that were audio recorded. An inductive thematic analysis approach was used.<br>• Country: England.<br>• Duration/year: Interviews between Nov 2015 – Apr 2016.<br>• Settings: OOH services.<br>• Population: GPs and NPs.<br>• Size: 30 (15 GPs and 15 NPs).	• 3-communicating decision stages: managing patient expectations, negotiating treatment, and safety netting.<br>• Several factors specific to OOH influenced antibiotic prescribing: lack of follow-up, access to GP records, consultation time and pressure, working contracts, patient–practitioner rapport, patient awareness, and implementation of feedback, audit, and supervision.<br>• NPs reported professional identity as a key influence on patient expectations, and the need for consistent prescribing was highlighted.<br>• Greater perceptions of accountability for prescribing and work to protocol among NPs compared with GPs.<br>• Delayed prescribing, peer discussion and education, and raising public awareness were reported as factors facilitating management decisions.<br>• Higher prescribing rates at weekends as an additional safety netting.
Zhu et al. (2021) [14]	• Investigate any changes in OOH antibiotic use prior to and during England's first wave of the COVID-19 pandemic.	• Method: Quantitative: Analysis of electronic-based prescribing data from NHS BSA.<br>• Country: England.<br>• Duration/year: Jan 2016 – Jun 2020.<br>• Settings: IH and OOH services.<br>• Population: Patients of all ages and genders.<br>• Size: Not reported.	• OOH made 3.3%-3.6% of primary care prescribing<br>• Before COVID-19, no significant change in the number of items prescribed OOH whereas the total prescribing volume and the percentage of broad-spectrum antibiotics continued to decrease within IH.<br>• Consistent decline in trimethoprim-to-nitrofurantoin ratio was observed in OOH and the proportion of broad-spectrum antibiotics was consistently higher in OOH.<br>• Prescribing volume peaked in December each year IH and OOH, and penicillins were prescribed the most in both settings.<br>• In March 2020, OOH prescribing volume started to fall; broad-spectrum prescribing rose in OOH and IH.<br>• Amoxicillin-clavulanic acid and doxycycline peaked in March to May 2020 in OOH, diverging from typical seasonal trends after COVID-19.

BEACH study: bettering the evaluation and care of health ; GP: general practice/practitioner; OOH: out-of-hours; URTIs: upper respiratory tract infections ;PAR: Participatory action research; GPC: general practice cooperative; IH: in-hours; ESAC: European surveillance of antimicrobial consumption; APQI: antibiotic prescribing quality indicators; iCAREdata: Improving Care and Research Electronic Data Trust Antwerp database; BAbAR: better antibiotic prescribing through Action Research; RTIs: respiratory tract infections; ICE: ideas, concerns and expectations; KIDPC: Kronoberg infection database in primary care; UTIs: urinary tract infections; NHS: National Health Service; CACE: complier average causal effect; ICPC: international classification of primary care; NHS BSA: National Health Service Business Services Authority; CCG: clinical commissioning group; GE: gastroenteritis; KUHR: control and payment of health reimbursement database; NorPD: Norwegian prescription database; CRP: C-reactive protein; DGP: daytime general practice; ARTIs: acute respiratory tract infections; RFE: reason for encounter; MAGNET: Melbourne East Monash general practice database NPs: nurse practitioners.

Search Strategy

Source: Ovid MEDLINE(R) ALL <1946 to May 09, 2022>
Date: May 09, 2022

Table S2. Medline search.

#	Search Terms	Results
1	Pre-hospital*.mp	5630
2	Prehospital*.mp	14944
3	Exp After-hours care/	2089
4	Out of hours.mp	2279
5	After hours.mp	3287
6	Outside of normal working hours.mp	116
7	1 or 2 or 3 or 4 or 5 or 6	24680
8	Prescrib*.mp	173493
9	Prescrip*.mp	139205
10	8 or 9	268279
11	Antimicrobial*.mp	207569
12	Antibiotic*.mp	428234
13	exp Anti-Bacterial Agents/	785111
14	11 or 12 or 13	1079160
15	7 and 10 and 14	120
16	Limited to 2017 onward	62

Source: Embase Classic+Embase <1947 to 2022 Week 18>
Date: May 09, 2022

Table S3. Embase search.

#	Search terms	Results
1	Pre-hospital*.mp	9976
2	Prehospital*.mp	20506
3	Exp After-hours care/	569
4	Out of hours.mp	4362
5	After hours.mp	2341
6	Outside of normal working hours.mp	214
7	1 or 2 or 3 or 4 or 5 or 6	35419
8	Prescrib*.mp	293787
9	Prescrip*.mp	317518
10	8 or 9	502722
11	Antimicrobial*.mp	295899

12	Antibiotic*.mp	918530
13	exp Anti-Bacterial Agents/	4257013
14	11 or 12 or 13	4448377
15	7 and 10 and 14	221
16	Limited to 2017 onward	96

Source: WoS Core Collection
Date: May 09, 2022

Table S4. Web of Science search.

#	Search Terms	Results
1	((TS=(pre-hospital* OR prehospital* OR "after hours" OR "out of hours" OR "outside of normal working hours")) AND TS=(prescrib* OR prescrip*)) AND TS=(antimicrobial* OR antibiotic* OR "anti-bacterial agents")	104
2	Limited to 2017 onward	52

Source: Ovid Emcare <1995 to 2022 Week 18>
Date: May 09, 2022

Table S5. Emcare search.

#	Search Terms	Results
1	Pre-hospital*.mp	3354
2	Prehospital*.mp	10423
3	Exp After-hours care/	213
4	Out of hours.mp	1474
5	After hours.mp	1015
6	Outside of normal working hours.mp	51
7	1 or 2 or 3 or 4 or 5 or 6	15564
8	Prescrib*.mp	77443
9	Prescrip*.mp	103546
10	8 or 9	147314
11	Antimicrobial*.mp	47985
12	Antibiotic*.mp	158799
13	exp Anti-Bacterial Agents/	618012
14	11 or 12 or 13	647691
15	7 and 10 and 14	91
16	Limited to 2017 onward	39

Source: Scopus
Date: May 09, 2022

Table S6. Scopus search.

#	Search Terms	Results
1	TITLE-ABS-KEY (*pre-hospital** OR *prehospital** OR *"after hours"* OR *"out of hours"* OR *"outside of normal working hours"*) AND TITLE-ABS-KEY (*prescrib** OR *prescrip**) AND TITLE-ABS-KEY (*antimicrobial** OR *antibiotic** OR *"anti-bacterial agents"*)	144
2	Limited to 2017 onward	72

Source: Cochrane library
Date: May 09, 2022

Table S7. Cochrane library search.

#	Search Terms	Results
1	Pre-hospital*	819
2	Prehospital*	2197
3	After-hours care	112
4	"out of hours"	203
5	"after hours"	141
6	"outside of normal working hours"	2
7	MeSH descriptor: [After-Hours Care] explode all trees	34
8	#1 OR #2 OR #3 OR #4 OR #5 OR #6 OR #7	2531
9	Prescrib*	30283
10	Prescrip*	18371
11	#9 OR #10	42263
12	Antimicrobial*	12580
13	Antibiotic*	35664
14	MeSH descriptor: [Anti-Bacterial Agents] explode all trees	12944
15	#12 OR #13 OR #14	48525
16	#8 AND #11 AND #15	37
17	in Cochrane Reviews, Cochrane Protocols, Trials and Editorials	35
18	Limited to 2017 onward	17

Source: **CINAHL Plus with Full Text** (EBSCOhost)
Date: May 09, 2022

Table S8. CINAHL search.

#	Search Terms	Results
1	"Pre-hospital*"	2942
2	(MH "Prehospital Care") OR "Prehospital*"	19415
3	"After-hours care"	88
4	""out of hours""	2585
5	""after hours""	28278
6	""outside of normal working hours""	44
7	S1 OR S2 OR S3 OR S4 OR S5 OR S6	51562
8	(MH "Prescribing Patterns") OR "Prescrib*"	73681
9	"Prescrip*"	83819
10	S8 OR S9	134070
11	"Antimicrobial*"	27383
12	"Antibiotic*" OR (MH "Antibiotics")	92323
13	"Anti-Bacterial Agents/" OR (MH "Antiinfective Agents")	37,786
14	S11 OR S12 OR S13	112403
15	S7 AND S10 AND S14	119
16	Limited to 2017 onward	63
